# Perspective about Cellulose-Based Pressure and Strain Sensors for Human Motion Detection

**DOI:** 10.3390/bios12040187

**Published:** 2022-03-22

**Authors:** Fevzihan Basarir, Joice Jaqueline Kaschuk, Jaana Vapaavuori

**Affiliations:** 1 Department of Chemistry and Materials Science, School of Chemical Engineering, Aalto University, FI-00076 Espoo, Finland; fevzihan.basarir@aalto.fi; 2 Department of Bioproducts and Biosystems, School of Chemical Engineering, Aalto University, P.O. Box 16300, FI-00076 Espoo, Finland; joice.kaschuk@ubc.ca; 3 Bioproducts Institute, Department of Chemical and Biological Engineering, The University of British Columbia, 2385 East Mall, Vancouver, BC V6T 1Z3, Canada

**Keywords:** cellulose, aerogel, hydrogel, foam, pressure sensor, strain sensor, resistive type, human motion detection

## Abstract

High-performance wearable sensors, especially resistive pressure and strain sensors, have shown to be promising approaches for the next generation of health monitoring. Besides being skin-friendly and biocompatible, the required features for such types of sensors are lightweight, flexible, and stretchable. Cellulose-based materials in their different forms, such as air-porous materials and hydrogels, can have advantageous properties to these sensors. For example, cellulosic sensors can present superior mechanical properties which lead to improved sensor performance. Here, recent advances in cellulose-based pressure and strain sensors for human motion detection are reviewed. The methodologies and materials for obtaining such devices and the highlights of pressure and strain sensor features are also described. Finally, the feasibility and the prospects of the field are discussed.

## 1. Introduction

Common prerequisites for wearable sensor systems are as follows: ultrathin, lightweight, flexible, stretchable, and conformable [1,2]. They can also form an interface with the human skin and capture biological signals accurately. By 2019, the wearable sensor market had reached approximately USD 10 billion with thousands of new patents, and hundreds of new companies and products [3,4].

Among sensor technologies, pressure and strain sensors have received great attention, owing to their possible use in next-generation human health monitoring including motion detection, machine interaction, soft robotics, and electronic skin [5,6]. In general, pressure and strain sensors convert external stimuli to electrical signals such as resistance, current, capacitance, and voltage. In this case, the external stimulus can be tensile or pressure, and in both cases, the sensor consists of electrodes and sensing elements. However, complex loading or shear can occur within the material while applying pressure or tensile load, respectively. The classification of pressure and strain sensors is based on their working principle, comprising piezoelectric, piezoresistive, and capacitive sensors. Out of these, piezoresistive wearable sensors have received considerably greater attention due to their simple structure, uncomplicated sensing mechanism, and low cost [7].

To exhibit piezoresistive features, wearable resistive pressure and strain sensors should include compressible, flexible and/or stretchable substrates, and electrically conductive materials. On top of that, they should encompass high sensitivity, fast response, wide detection range, skin friendliness, biocompatibility, and endurance over multiple detection cycles [8,9]. Silicone rubber polymers such as polydimethylsiloxane (PDMS) have been widely used as substrates for pressure and strain sensors due to their flexibility and stretchability [10]. However, these materials do not fulfill all the requirements, such as skin affinity and the potentiality of production from renewable raw materials sources.

So, cellulosic materials, more specifically nanocellulose, have emerged as a promising candidate for the development of such sensors. They have outstanding properties such as skin affinity, low-cost production, non-toxicity, renewability, biodegradability, and flexibility [11]. In addition, these materials present a great opportunity for obtaining substrates with high mechanical strength, large surface area, high aspect ratio, and more importantly ample range of tailorability due to the possible hydroxyl groups modifications [12]. However, nanocellulose materials need to be tuned to contain the required features of a pressure and strain sensor. For instance, nanocellulose materials suffer from low electrical conductivity and some structures present low resistance to compression or stretching. To solve these issues, composites and 3D structures have been considered for the development of high-performance sensors.

**Figure 1:**
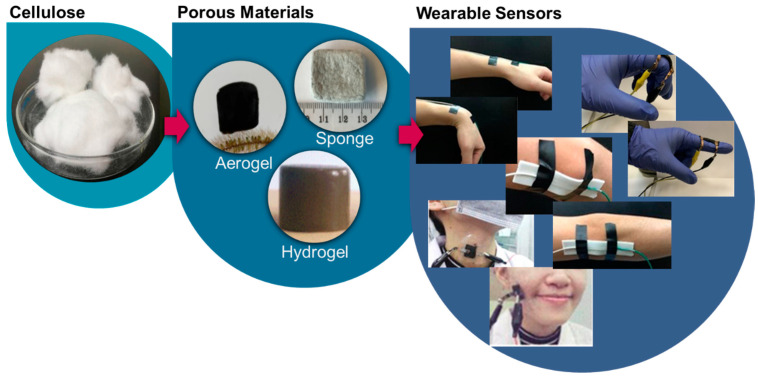
Pressure and strain sensors produced with nanocelluloses for human motion monitoring. Cellulose can be used to produce aerogels (Reproduced with permission from [13] Copyright 2021, American Chemical Society), sponges (Reproduced with permission from [14] Copyright 2019, American Chemical Society), and hydrogels (Reproduced with permission from [15] Copyright 2021, Elsevier), which can be converted into wearable sensors that detect a variety of movements such as wrist, elbow (Reproduced with permission from [16] Copyright 2020, Elsevier), and finger bending (Reproduced with permission from [17] Copyright 2018, Elsevier), throat movement, and facial expressions (Reproduced with permission from [18] Copyright 2020, Springer).

In this regard, we present some of the requirements for producing nanocellulose 3D network structures (hydrogels and air-porous materials) with features beneficial for pressure and strain sensors (Figure 1). We also review recent advances in cellulose-based pressure and strain sensors for human motion detection. First, we summarize how it is possible to produce hydrogels and air-porous materials, and how these materials can be converted to pressure and strain sensors. Additionally, we identify the desired characteristics of such devices and highlight the promise of cellulosic materials for the development of biobased sensors. Finally, we discuss the outlook on the future of this technology.

## 2. Cellulosic Porous Materials

**Figure 2:**
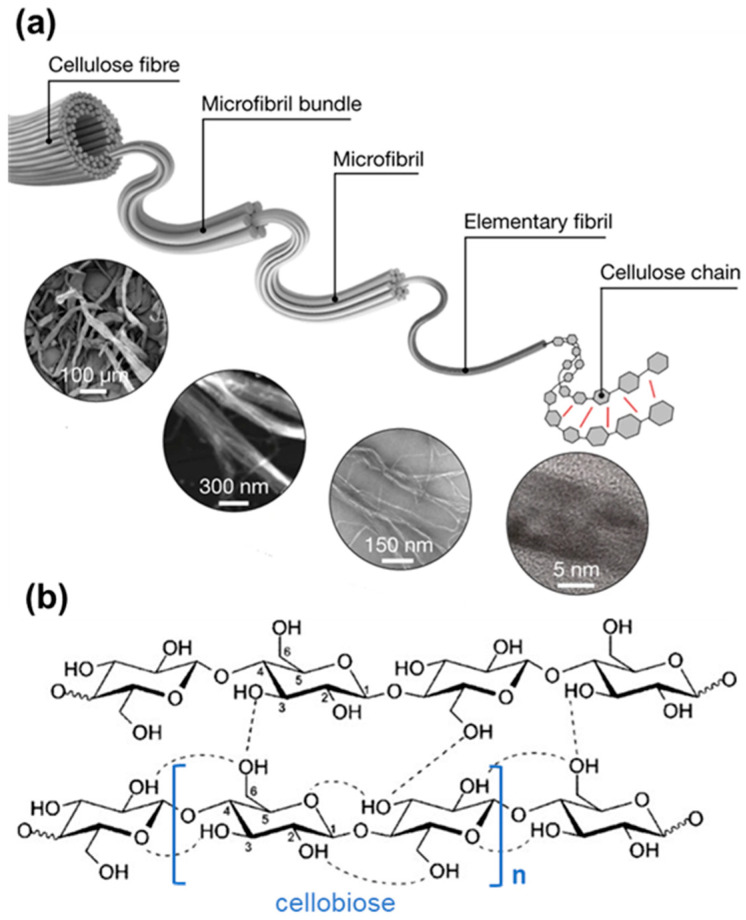
(**a**) Hierarchical structure of fibrillated cellulose (Reproduced with permission from [22] Copyright 2021, Springer) and (**b**) Chemical structure of cellulose and its hydrogen bonds.

Cellulose [(C_6_H_10_O_5_)*_n_*] is a homopolysaccharide consisting of a structural unit called cellobiose linked by β-(1-4) glycosidic bonds (β-D-glucan) (Figure 2) [19]. Cellobiose, consecutively, is composed of two glucose molecules containing three hydroxyl groups each [19,20]. These hydroxyl groups are responsible not only for the high hydrophilicity of cellulose but also for its crystalline/non-crystalline regions and hierarchical organization due to the van der Waals forces and the hydrogen bonds they contribute to [21]. In nature, cellulose fibers are organized in such a way that it is possible to extract materials with different diameters varying from micrometers (100 µm) to nanometers (2–4) in size [22].

To obtain nanoscale cellulose materials from renewable sources, such as tree and crop wastes, the macroscopic fibers (tens of microns in diameter) are broken down by different chemical and mechanical treatments [23] and are commonly referred to as nanocellulose [24]. Usually, nanocellulose materials produced by mechanical and/or enzymatic treatments are called cellulose nanofibrils (CNF) and those produced by chemical treatment (usually acids) are called cellulose nanocrystals (CNC) [20,23,25]. CNFs contain both non-crystalline and crystalline regions, while CNCs present mostly crystalline regions. This not only brings differences in length and diameter between CNFs (submicron to tens of microns in length, diameters of several nanometers to several microns) [23] and CNCs (100 to 200 nm in length and 10 to 30 nm in diameter) [23], but also in the fibril’s flexibility. CNFs are more flexible with a higher capacity for producing a stable 3D-structured network, while CNCs require some crosslinking agents to achieve this 3D-structured network [26]. Additionally, nanocellulose can also be produced by bacterial genera such as Agrobacterium, Aerobacter, Achromobacter, Sarcina, Acetobacter, Rhizobium, Salmonella, and Azotobacter [27]. This type of bacterial cellulose (BC) is a nanoscale network with pure cellulose fibers between 20 and 100 nm with properties depending on the growth media conditions, bacteria employed, fermentation conditions, and nutrient sources [28]. In summary, for produced nanocellulose from different bacteria with a distinct mechanism, the nanocellulose presents different yields, degrees of polymerization and crystallinity [29].

With crosslinking reactions or self-entanglement between adjacent nanocellulose chains, a 3D network nanocellulose structure can be designed [30]. When the process occurs by chemical reaction, the functional groups of the cellulose, such as hydroxyl groups, are linked by small molecules. Self-entanglement, on the other hand, takes place by physical interactions, such as hydrogen bonds. In both cases, the cellulose chains are intertwined producing a porous network structure [31]. Both hydrogel and air-porous materials are similar in terms of this porous structure; however, when water is the continuous fluid in the matrix, we have a hydrogel, and when it is air, we have air-porous materials [30]. Air-porous materials can be further classified as foams, sponges, and aerogels. Foams and sponges are produced by dispersing gases (air) inside of liquid and solid-state materials, and they have pore diameters greater than 50 nm and macro-sized pores, respectively [32]. Aerogels are solid materials with nano-porous networks which can be obtained through lyophilizing hydrogels [32]. In this process, the hydrogel is frozen and the water molecules can be removed by freeze drying (low temperature, low pressure) or via solvent exchange and subsequent critical point drying (CPD, high temperature, high pressure) [31].

**Figure 3:**
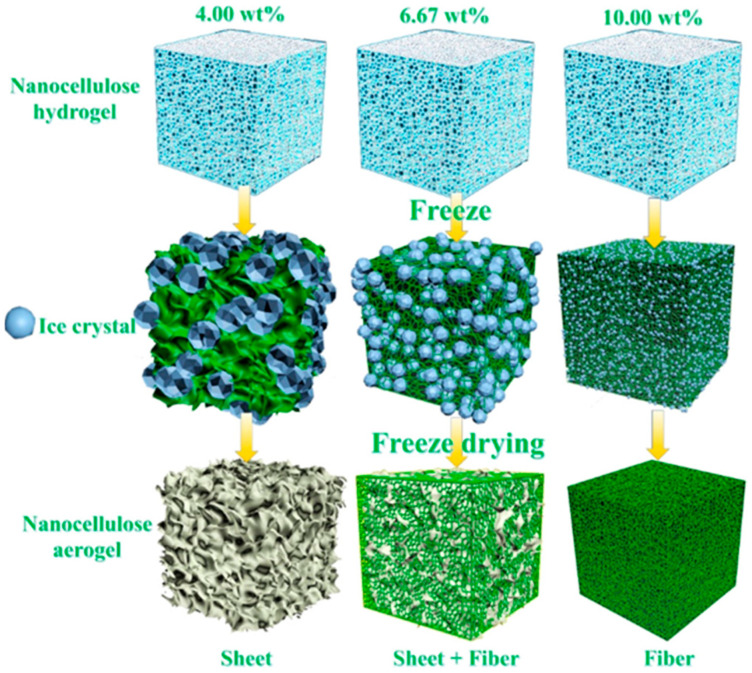
Schematic of producing aerogels from hydrogel using different concentrations of CNF (Reproduced with permission from [33] Copyright 2020, American Chemical Society).

Additionally, the structure of air-porous materials and hydrogels is dependent on the initial concentration of nanocellulose, as observed by Huang and collaborators [33]. They showed that through the freezing step of hydrogel production, the size of the ice created is dependent on the nanocellulose concentration and impacts the pore sizes of the produced aerogels [33]. So, the greater the concentration of nanocellulose, the greater the fiber interlacement and the smaller the size of the ice crystals, and consequently the smaller the pores (Figure 3) [33]. These changes in the concentration and consequently the size of the pores also result in alterations in the mechanical properties (compression) of these materials. When using the same source of nanocellulose, an increase in the concentration leads to an increase in Young’s modulus, in other words, the materials become more resistant to compression [34,35].

Another feature to be considered in these materials is the type of nanocellulose used to produce hydrogels and aerogels. For instance, physically crosslinked CNC aerogels (crosslinking: CaCl_2_) have a lower compressive strength than CNF aerogels (crosslinking: CaCl_2_), and the same behavior is expected for hydrogels [31,36]. This is attributed to the structural differences between CNF and CNC, once CNF presents a longer chain and less crystallinity, which results in a more entangled structure compared to the short and highly crystalline CNCs [31].

It is worth highlighting that the inherent tunability of the mechanical properties is highly useful for having sensors of different sensorial ranges. For instance, weak pressing/tensile forces can be detected by highly sensitive sensors. Besides, the 3D structures can be extremely beneficial for sensor design since additives or blending materials can be implemented producing better electrical properties and multiple responses. Another approach is the combination of nanocellulose with another polymer, also called biocomposites. In general, the production of these biocomposites is intended to increase the resistance to compression and tension. In the literature, it is possible to find examples of biocomposites produced from CNC and CNF with polymeric matrices, such as polydimethylsiloxane (PDMS), waterborne polyurethane, alginate, and polyvinyl alcohol (PVA) [37]. In most of the cases, incorporation happens by infiltration of the desired polymer, filling the cellulosic porous materials. So, a better mechanical property is achieved [37].

To implement the piezoresistive sensing function of these materials, electrical conductivity needs to be implemented. In the case of nanocellulose materials, it is necessary to introduce conductive materials in the structure of air-porous materials and hydrogels. Among the most common materials to produce conductivity are carbon nanotubes, graphene, graphite, and metallic nanowires such as silver nanowires [37]. A successful example is the production of honeycomb cellulose nanofiber aerogel with different concentrations of carbon nanotubes by Wang and collaborators [38]. In this case, the carbon nanotubes were incorporated together with PDMS with different concentrations and by increasing the concentration of the carbon nanotubes from 10 to 50 wt.%, the conductivity increased from 1 × 10^−4^ to 4.32 S.m^−1^. In addition to establishing the influence of concentration, they also realized that conductivity increases when the particles are better distributed and aligned [38]. In the next sections, we summarize how air-porous materials and hydrogels can be used as substrates for pressure and strain sensors.

## 3. Working Principle of Piezoresistive Sensors

### 3.1. Pressure Sensor

**Figure 4:**
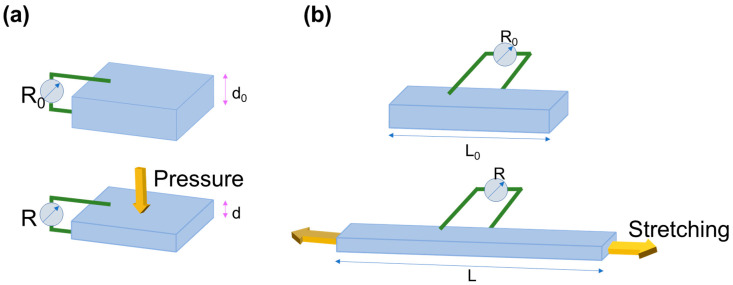
Schematic demonstration of working mechanism of (**a**) pressure sensor and (**b**) strain sensor.

Piezoresistivity is known as the electrical resistance change of a material caused by its structural deformation. As shown in Figure 4a, the principle working mechanism of a pressure sensor relies on the resistance change due to the applied pressure. Key performance parameters for the sensor could be defined as resistance change, compression strain, and sensitivity. Strain, resistance, and sensitivity can be expressed as ε = (d_0_ − d)/d_0_, ΔR/R_0_ = (R − R_0_)/R_0_, S = (ΔR/R_0_)/ε, respectively, where d_0_ is the initial thickness of the sensor, d is the thickness of the sensor under compression, R_0_ is the initial resistance of the sensor, and R is the resistance of the sensor under compression.

### 3.2. Strain Sensor

The piezoresistive strain sensor working principle depends on the resistance change of the sensor due to the applied strain, as demonstrated in Figure 4b. The strain can be evaluated as follows, ε = (L − L_0_)/L_0_, where L_0_ is the initial length of the sensor, and L is the length of the sensor under stretching. The other parameter is the resistance change which can be determined as follows, ΔR/R_0_ = (R − R_0_)/R_0_, where R and R_0_ are the resistances of the samples without and with applied strain, respectively. Besides, the sensitivity of the sensors is defined by the gauge factor, which can be quantified as GF = (ΔR/R_0_)/ε.

However, it is worth noting that the sensitivity of the pressure and strain sensors can be tuned by the concentration of the conducting materials, which is related to the percolation threshold.

## 4. Cellulosic Air-Porous Materials for Pressure Sensors

**Figure 5:**
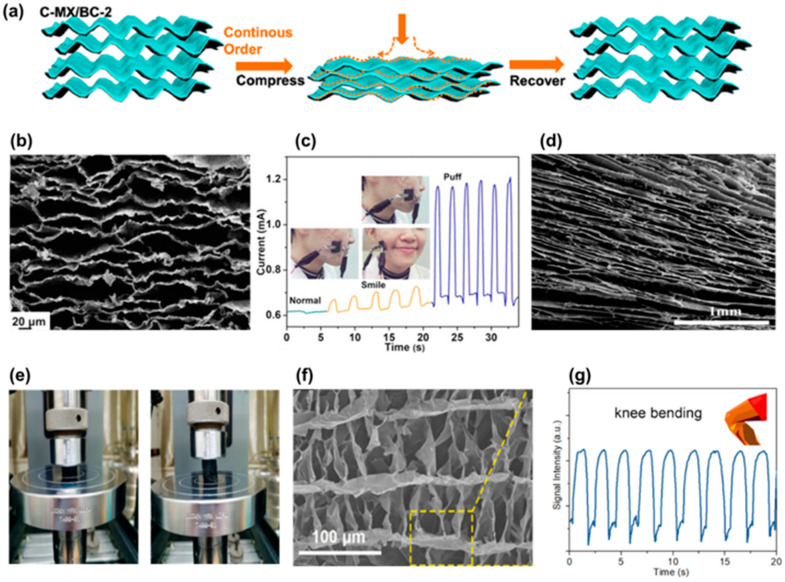
Properties of the Ti_3_C_2_/BC aerogel sensor: (**a**) Compressibility and elastic mechanisms of the aerogel, (**b**) cross-section SEM image of the aerogel, and (**c**) current signals from face expressions ((**a**–**c**), Reproduced with permission from [39] Copyright 2019, American Chemical Society). Properties of the waterborne polyurethane/cellulose nanocrystal/carbon nanotubes/graphene aerogel sensor: (**d**) cross-section SEM image of the aerogel and (**e**) photographs of the aerogel during compressing and releasing process ((**d**,**e**), Reproduced with permission from [13] Copyright 2021, American Chemical Society). Properties of the carbonized BC/CNF aerogel sensor: (**f**) cross-sectional morphology of the sensor and (**g**) application of the wearable sensor to detect knee bending ((**f**,**g**), Reproduced with permission from [40] Copyright 2021, American Chemical Society).

When an external force is applied to a pressure sensor, changes are observed in the electrical properties of the sensor [14]. Thus, elastic air-porous materials are very promising for applications in wearable sensors, since they can provide better compression/recovery properties. This effect was demonstrated by Chen et al. [39], who produced a lightweight aerogel-type resistive sensor consisting of Ti_3_C_2_ (MXene—materials of 2D transition metal carbides/nitride) sheets and BC fibers. The aerogel was produced by first directional freezing, followed by freeze drying, and carbonization [39]. Even though the BC was completely converted to carbon through the carbonization process, the BC fibers provided binding sites between the MXene sheets, creating a continuous and wave-shaped lamellar macrostructure (Figure 5a,b). Comparing with the bare MXene materials, the oriented alignment of these lamellae structures not only provided superior compressibility and elasticity, but also good retention of the performance that lasted for 100.000 cycles at 50% strain.

Another feature observed was the improvement in the efficient stress transfer along with the entire lamellar structure. The sensor was highly sensitive to compression strain and can distinguish different levels of compression strains. In addition, the measured current intensity had a linear relationship with the applied pressure, demonstrating a sensitivity of 12.5 kPa^−1^. The sensor provided rapid response (167 ms) and recovery (121 ms) abilities and it also easily detected facial expressions, such as puffing and smiling (Figure 5c).

Consequently, to utilize the benefit of 2D nanomaterials, Zhai and colleagues fabricated graphene-based highly sensitive sensors which also comprised waterborne polyurethane, cellulose nanocrystal, carbon nanotubes, and graphene [13]. This air-porous material showed pore sizes of 110–180 μm and a 3D lamellar structure (Figure 5d). The piezoresistive characteristics of this composite presented a detection limit of 0.112 kPa and a high sensitivity of 0.25 kPa^−1^ in the low-pressure region (0 to 4.2 kPa) and 0.048 kPa^−1^ in the high-pressure region (4.2–10 kPa). In addition, this sensor showcased excellent mechanical performance with only minor damage after 800 cycles and excellent resistance response stability (Figure 5e).

Nanocellulose can also be a source for carbonized materials, as demonstrated by Chen and collaborators [40]. They produced sheets of carbonized bacterial nanocellulose (CBC) and incorporated them into CNF suspensions. After that, they prepared CBC-CNF aerogels via directional ice-templating and freeze drying [40]. The obtained aerogel showed a porous structure with well-aligned channels and the carbonized flakes were found to be dispersed onto the CNF-based pore walls (Figure 5f). Interestingly, the pressure sensors demonstrated tunable sensitivity (0.003–0.358 kPa^−1^) by simply changing the CNF concentration. The response time was measured to be 50 and 110 ms for the pressure loading and unloading, respectively. The low detection limit of the sensor was ~2.5 Pa. The sensor indicated excellent durability and reliability over 10,000 loading−unloading cycles. Finally, the sensor was used successfully in the detection of wrist and knee bending (Figure 5g).

**Figure 6:**
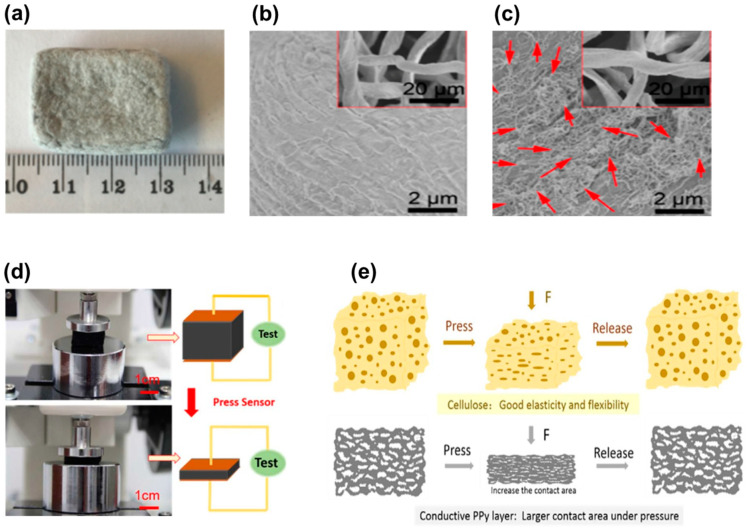
Properties of the cellulose/MWCNT sponge: (**a**) macro image of the sponge, (**b**) SEM image of the cellulose sponge without MWCNT, and (**c**) SEM image of the cellulose sponge with MWCNT ((**a**–**c**), Reproduced with permission from [14] Copyright 2019, American Chemical Society). Properties of the cellulose/PPy sponge: (**d**) macro images of pressure and deformation test of the sponge and (**e**) schematic illustration of compressing and recovering process of cellulose sponge and PPy cellulose sponge ((**d**,**e**), Reproduced with permission from [41] Copyright 2017, Elsevier).

However, not only aerogels can be used for the fabrication of pressure sensors, as demonstrated by Zhang and collaborators [14]. In their work, they fabricated a sponge-based pressure sensor where multiwalled carbon nanotubes (MWCNT) were dispersed in cotton cellulose solution (sodium hydroxide (NaOH): urea), which was poured into a cube mold, and immersed in a water bath. A macro image of a typical sensor with 10% MWCNT is shown in Figure 6a. The morphology of the cellulosic sponge without MWCNT is exhibited in Figure 6b, which shows the smooth and scaly structure. The addition of MWCNT (10%) resulted in the uniform deposition of the nanotubes on the cellulose fibers (Figure 6c). Increasing the pressure on the sensor had a linear change on the resistance. The sensor was also subjected to finger pressing and the response was rapid and stable, with a small hysteresis.

Moreover, sponges using cellulose/NaOH/urea solutions can also be prepared by the freeze-drying method accompanied by in situ chemical oxidative polymerization of pyrrole [41]. In this case, the composite sponge also presented good electrical/mechanical properties for sensor development. Thus, Figure 6d shows how this cellulosic sponge sensor presents good mechanical properties under pressure. As the pressure applied to the sponge increases, the layered structure is compressed, and the electrical resistance is reduced prevenient from the increase in the contact area of the conductive polypyrrole layer and possible increased electron transport channels (Figure 6e) [41].

## 5. Cellulosic Hydrogels for Pressure Sensors

**Figure 7:**
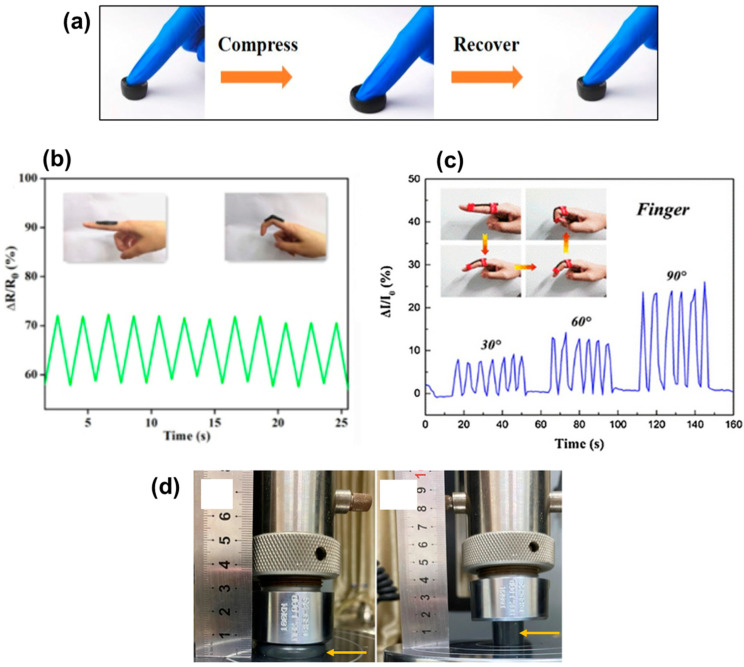
Properties of PC/CNF/LC hydrogel sensor: (**a**) demonstration of elasticity of sensor and (**b**) finger bending response of the sensor ((**a**,**b**), Reproduced with permission from [42] Copyright 2021, Elsevier). Properties of CNF/AgNP/PAM hydrogel sensor: (**c**) finger bending response of the sensor (Reproduced with permission from [15] Copyright 2021, Elsevier). Properties of CNC/MXene hydrogel sensor: (**d**) mechanical property of the sensor (Reproduced with permission from [43] Copyright 2021, Springer).

As mentioned before, hydrogels are porous materials where water is the continuous fluid, and they are also good materials for sensor applications. For instance, Li et al. [42] produced a sensor from a hydrogel containing polyvinyl alcohol (PVA), carboxymethyl chitosan, CNF, and lignin-based carbon (LC). In this hydrogel, LC acted as the conductive filler in the material [42]. Here, the crosslinking bonds between the components resulted from the hydrogen bonds between the hydroxyl, amino, and carboxyl groups. The final hydrogel presented compressible and elastic properties suitable for devices where recovery is required (Figure 7a). The sensitivity and stability performance of the sensors was evaluated by increasing the compression from 10 to 70%, leading to an increase in the R/R_0_ of cyclic compression. When tested as a sensor attached to the index finger, this hydrogel demonstrated fast, stable, and reversible behavior upon bending the finger (Figure 7b).

Then, Wang et al. [15] prepared hydrogels using TEMPO-oxidized CNF and silver nanoparticles both crosslinked with polyacrylamide (PAM) network. The excellent mechanical performance and elasticity of this hydrogel were suitable for the production of skin-like hydrogel sensors [15]. The sensor showed a steady response to regular pressure and the sensitivity was calculated to be 9.5 and 1.6 kPa^−1^ for the pressure range of 0–5 kPa and 5–50 kPa, respectively. The real-time current changes were investigated via attaching the hydrogels on a finger and bending it with different angles (Figure 7c), demonstrating the sensitivity of the sensor to bending the finger.

Pi et al. [43] combined CNCs and MXene to prepare nanosheets, which were used as fillers in crosslinked PVA and polyacrylamide hydrogel matrix [43]. The obtained hydrogel is elastic and recovers after releasing the stress, as shown in Figure 7d. The hydrogel could hold 1.1 MPa compression, indicating excellent compressibility. However, the authors did not further investigate the pressure sensor measurements and applications.

The explorations and studies applying cellulosic materials for pressure sensors are still quite limited, as can be observed from Table 1. However, this does not mean that it is not highly important to enhance and develop this area. It is still necessary to enhance the performance, stability, repeatability, and especially the sensitivity of such sensors. As can be seen in Table 1, the pressure sensors present higher performance particularly in terms of sensitivity in the low-pressure region compared to the commercial sensors used in medical applications (fluid monitoring applications, dialysis machines, and physical therapy equipment).

## 6. Cellulosic Air-Porous Materials for Strain Sensors

In this section, we will describe how cellulose can be applied for strain sensors. However, before we start, it is good to make clear that one of the differences between pressure sensors and strain sensors regards how the external force is applied. Strain sensors have an electrical properties response from a tensile force. Taking into account that the tensile stretchability of air-porous materials tends to be fairly limited, there are very few works about using air-porous materials in this function.

**Figure 8:**
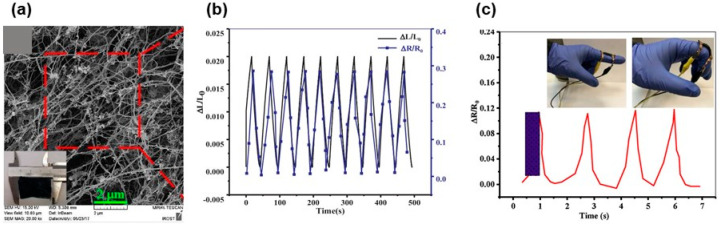
Properties of BC/MMWCNT aerogel sensor: (**a**) morphology of the aerogel, (**b**) cycling tensile loading of the aerogel, and (**c**) relative resistivity variation during finger bending ((**a**–**c**), Reproduced with permission from [17] Copyright 2018, Elsevier).

Taking advantage of the growing process of the BC, Hosseini and coauthors [17] produced an aerogel strain sensor containing MWCNTs. In summary, the nanotubes were added to the grown media, and the produced BC composite hydrogel was dried in supercritical CO_2_ [17]. The BC nanofibers and MWCNTs intertwine, where the MWCNTs adhered strongly to the surface of BC (Figure 8a) providing the electrical properties for the aerogel. As shown in Figure 8b, ΔR/R_0_ (R_0_ represents the initial resistance and ΔR symbolizes the difference between the measured and initial resistance) increased upon applying stress and then reduced when the stress was released which was attributed to the decreasing and increasing interparticle distances, respectively. A gauge factor of 21 and response time of 390 ms were reported for the sensor. In addition, to monitor the finger motions, the sensor was attached to a latex glove, as shown in Figure 8c; resistance increase, and a decrease was observed upon bending and unbending of the finger.

## 7. Cellulosic Hydrogels for Strain Sensors

**Figure 9:**
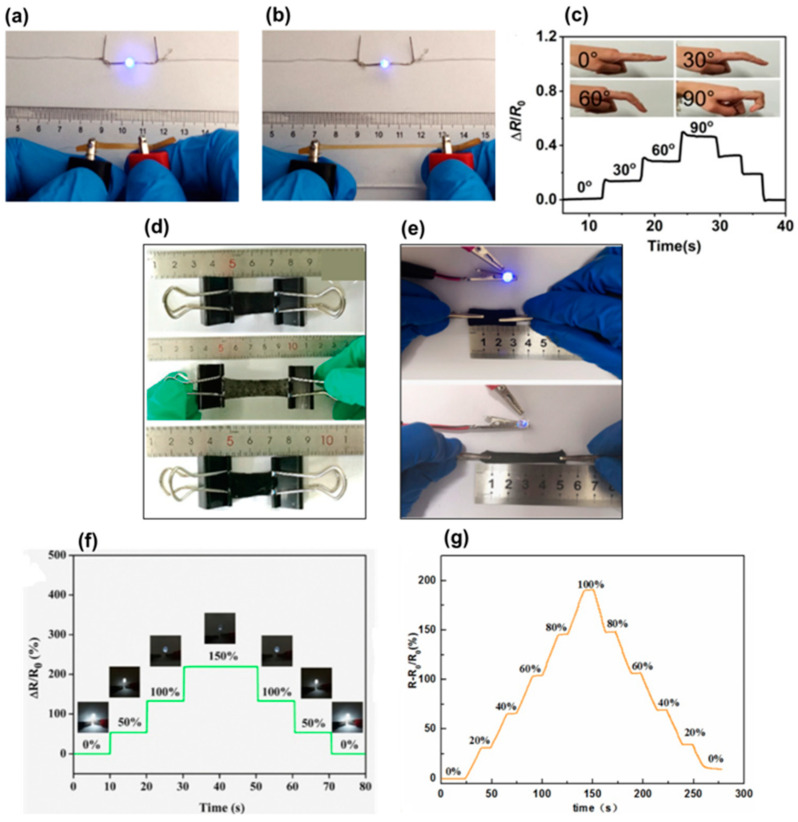
Properties of carboxymethyl cellulose Fe^3+^/polyacrylamide double network hydrogel sensor: (**a**) conductive property of the sensor, (**b**) decrease in the brightness of the bulb with increased strain, and (**c**) detection of finger bending at different angles (0°, 30°, 60°, and 90°) and the corresponding ΔR/R_0_ ((**a**–**c**), Reproduced with permission from [18] Copyright 2020, Springer). Properties of PVA/SA/BC/MCC hydrogels: (**d**) optical images of before, during, and after stretching (Reproduced with permission from [44] Copyright 2020, Wiley). Properties of PC/CNF/LC hydrogel sensor: (**e**) conductivity and (**f**) ΔR/R_0_ as a function of the applied strain ((**e**,**f**), Reproduced with permission from [42] Copyright 2021, Elsevier). Properties of the cellulose Zn^2+^/PVA hydrogel sensor: (**g**) time profile of relative resistance changes with consecutive applied strain (Reproduced with permission from [45] Copyright 2022, Elsevier).

Zhang et al. [18] developed dual physical crosslinking between carboxymethyl cellulose-Fe^3+^/polyacrylamide (CMC-Fe^3+^/PAAm) producing a double network hydrogel [18]. This hydrogel exhibited excellent conductivity and the LED indicator was lit (Figure 9a); however, upon stretching the hydrogel from 0 to 300%, the light intensity of the LED gradually diminished (Figure 9b). The ΔR/R_0_ linearly increased with the strain, providing a gauge factor of 1.99 and 4.02 for 0–50% and 50–600% strain range, respectively. In addition, the hydrogel demonstrated a fast response time of 260 ms. The ΔR/R_0_ change showed cyclic behavior when the hydrogel was subjected to loading–unloading at a strain of 100% with different speeds, indicating that the hydrogel was able to monitor the deformation from 1 to 500% with excellent repeatability. The hydrogel sensor was also attached to the index finger and the ΔR/R_0_ was increased with increasing the bending angle, as shown in Figure 9c.

In addition, Huang et al. [44] fabricated PVA/sodium alginate-based double physical crosslinked hydrogels reinforced with BC, and containing MWCNTs and carbon black as the conductive elements [44]. Figure 9d shows the hydrogel sensor before, during, and after stretching. A monotonic increase in the ΔR/R_0_ could be monitored with a strain range of 0–200%, and ΔR/R_0_ was obtained at the maximum strain (200%), indicating the excellent response of the sensor to applied strain. In addition, the gauge factor was calculated to be 0.725, 2.216, and 5.01 for a strain range of 0–60%, 60–145.2%, and 145.2–200%, respectively. The intensity of resistance signals increased gradually upon stretching to 10%, 50%, 100%, and 150%, and the ΔR/R_0_ was almost the same value under repeated stretching of each strain, indicating the repeatability and stability of the sensor.

Similar to the hydrogel pressure sensor already described, Li et al. [42] tested the hydrogel containing PVA, carboxymethyl chitosan, and CNF carbonized lignin for strain sensors. As shown in Figure 9e, the brightness of the diode bulb decreased (the sensor and the diode bulb connected by copper wires) with increasing strain, indicating the conductivity of the hydrogel. In addition, the ΔR/R_0_ showed a tendency of increasing gradually with stretching, and the resistance almost recovered to the initial value once the strain was released (Figure 9f). The resistance of the hydrogel changed steadily and responded quickly during the stretching process. These results revealed high strain sensitivity and excellent electrical stability.

In the study developed by Hu and collaborators, they prepared PVA-based hydrogels reinforced by CNF containing ZnSO_4_ through a one-pot simple freezing–thawing method [45]. The resulting hydrogel sensor presented a good sensing performance as shown in Figure 9g, providing a fast and reversible response upon changing the strain amount. For another example, Qin et al. [46] produced a hydrogel via a combination of BC/sodium alginate/polyacrylamide with the polyaniline through multiple intermolecular interactions. The relative resistance changes of this sensor indicated a fast response, reversibility, and repeatability during stretching and releasing cycles with 100%, 150%, and 200% strain.

The low elasticity of cellulosic materials makes it harder to apply these materials for strain sensors, as demonstrated by the lower number of research reports collected in Table 2. However, these could be an opportunity for sensors that require low strain changes for detection. Finally, we can say that comparing these sensors with commercial ones used in medical applications (syringe pumps and kidney dialysis machines), we can observe better performances typically in terms of gauge factor.

## 8. Outlook and Conclusions

In this work, we summarized the findings of pressure/strain sensors using nanocellulose-based hydrogels and air-porous materials. It was demonstrated how this extraordinary biomaterial can be tuned to successfully produce sensors with notable performance and sensitivity. However, we conclude that the field is still under development, the key question being optimization of microstructures and porosity of elastic materials with multiple functions for high-performance sensors. One of the main reasons for the relatively slow pace of progress could be that nanocellulose with good quality is not easily commercially accessible. The other reasons are the low elasticity and the non-existent solubility of nanocellulose in many common solvents, the latter limiting the manipulation of nanocellulose. Nevertheless, we believe that once optimized, 3D structures such as hydrogels and air-porous materials of nanocellulose could have a bright future in the sensor field.

In summary, the full spectrum of different air-porous materials that can be fabricated out of cellulose has not yet been exploited—for instance broader pressure ranges could be covered by sensors with optimized mechanical properties. Self-assembly of 2D nanomaterials (MXene and graphene) with nanocellulose demonstrated promising results especially for air-porous pressure sensors; however, only a limited number of studies have been carried out. Hence, it is necessary to investigate further the effect of different 2D nanomaterials and freezing conditions on the sensory properties.

Furthermore, another beneficial aspect specifically related to air-porous materials, the light total weight of the sensors, could be explored more. Since air-porous nanocellulose materials can reach densities as low as 0.01 g/cm^3^, they are ideally suited for the design of lightweight functional sensors. Besides, long-term plasticity is an important issue to be tested for the air-porous materials (aerogel and sponge). Concerning hydrogel-based sensors, long-term stability is an important issue since they may lose their water with time, which may deteriorate the sensing performance. Self-assembly of nanocellulose with other conductive nanomaterials such as silver nanowires, copper nanowires, and gold nanoparticles should also be considered. Overall, the design of skin friendly/conformal and high-performance wearable pressure/strain sensors for health care monitoring built with different nanocellulose-based materials are vast and promising.

## Figures and Tables

**Table 1:** Properties of the pressure sensors prepared with cellulosic materials.

Material Type	Conductive Material	Cellulose Type	Sensitivity (kPa^−1^)	Detection Limit (kPa)	Response (ms)	Recovery (ms)	Cyclic Stability	Human Body Monitoring	Ref.
Air-porous	MXene	Bacterial cellulose	12.5	-	167	121	100,000 (50% strain)	Finger bending Wrist bending Elbow bending Face expression Arm pulse Jugular venous pulse	[39]
	CNT/Graphene	CNC	0.25	0.112	-	-	-	Finger bending Wrist bending Elbow bending Squatting Walking Running	[13]
	Carbonized bacterial cellulose	TEMPO CNF	0.003–0.358	0.0025	50	110	10,000	Wrist bending Knee bending Finger touch Breath blow	[40]
	Multiwalled carbon nanotube (MWCNT)	Cotton balls	0.0159–0.0197	-	20	20	-	Finger compression	[14]
	Polypyrrole	Cellulose microcrystalline	58.9	-	-	-	10	-	[41]
Hydrogel	Lignin carbon	CNF	-	-	-	-	-	Finger bending Elbow bending Palm gripping	[42]
	Silver nanoparticle	TEMPO CNF	9.5	-	-	-	1000	Face expression Elbow bending Neck forward Walking Jumping	[15]
	MXene	CNC	-	-	-	-	-	-	[43]

**Table 2:** Properties of the strain sensors prepared with cellulosic materials.

Material Type	Conductive Material	Cellulose Type	Gauge Factor	Response (ms)	Cyclic Stability	Human Body Monitoring	Ref.
Air-porous	Multiwalled carbon nanotube (MWCNT)	Bacterial cellulose	21	390	1000	Finger bending	[17]
Hydrogel	Na^+^ Fe^3+^	Carboxymethyl cellulose	1.99 (0–50%) 4.02 (50–600%)	260	-	Finger bending Wrist bending Elbow bending Swallowing Speaking	[18]
	MWCNT Carbon black	Bacterial cellulose	0.725 (0–60%) 2.216 (60–145.2%) 5.010 (145.2–200%)	-	-	Wrist bending Elbow bending	[44]
	Carbonized lignin	CNF	-	-	-	Finger bending Wrist bending Elbow bending	[42]
	Zn^2+^	TEMPO CNF	1.70	-	500	Finger bending Wrist bending Elbow bending Knee bending Neck movement Speaking	[45]
	Polyaniline	Bacterial cellulose	0.85	560	200	Finger bending Wrist bending Elbow bending Knee bending	[46]
